# Nanoplastic Transport in Soil via Bioturbation by *Lumbricus terrestris*

**DOI:** 10.1021/acs.est.1c05614

**Published:** 2021-12-08

**Authors:** Wiebke Mareile Heinze, Denise M. Mitrano, Elma Lahive, John Koestel, Geert Cornelis

**Affiliations:** † Department of Soil and Environment, Swedish University of Agricultural Sciences, Box 7014, 75007 Uppsala, Sweden; ‡ Department of Environmental Systems Science, ETH Zurich, Universitätsstrasse 16, 8092 Zürich, Switzerland; § UK Centre for Ecology and Hydrology, Benson Lane, Crowmarsh Gifford, Wallingford, OX10 8BB, United Kingdom; ∥ Agroscope − Standort Reckenholz, Soil Quality and Soil Use, Reckenholzstrasse 191, 8046 Zürich, Switzerland

**Keywords:** Microplastic, transport, fate, exposure, X-ray computed tomography, earthworms

## Abstract

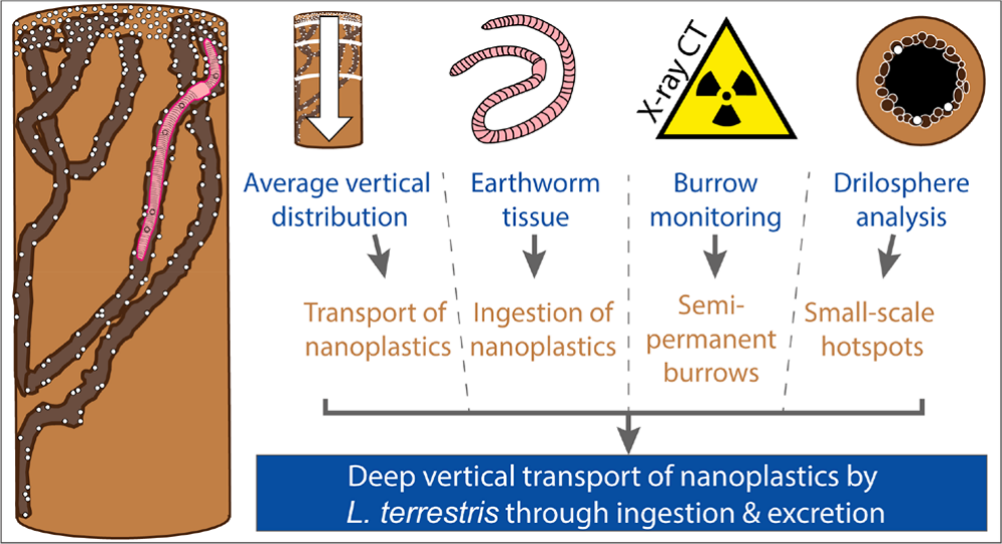

Plastic pollution is increasingly perceived as an emerging threat to terrestrial environments, but the spatial and temporal dimension of plastic exposure in soils is poorly understood. Bioturbation displaces microplastics (>1 μm) in soils and likely also nanoplastics (<1 μm), but empirical evidence is lacking. We used a combination of methods that allowed us to not only quantify but to also understand the mechanisms of biologically driven transport of nanoplastics in microcosms with the deep-burrowing earthworm *Lumbricus terrestris*. We hypothesized that ingestion and subsurface excretion drives deep vertical transport of nanoplastics that subsequently accumulate in the drilosphere, i.e., burrow walls. Significant vertical transport of palladium-doped polystyrene nanoplastics (diameter 256 nm), traceable using elemental analysis, was observed and increased over 4 weeks. Nanoplastics were detected in depurated earthworms confirming their uptake without any detectable negative impact. Nanoplastics were indeed enriched in the drilosphere where cast material was visibly incorporated, and the reuse of initial burrows could be monitored via X-ray computed tomography. Moreover, the speed of nanoplastics transport to the deeper soil profile could not be explained with a local mixing model. Earthworms thus repeatedly ingested and excreted nanoplastics in the drilosphere calling for a more explicit inclusion of bioturbation in nanoplastic fate modeling under consideration of the dominant mechanism. Further investigation is required to quantify nanoplastic re-entrainment, such as during events of preferential flow in burrows.

## Abstract 2

Risk assessment of nanoplastics in soils should consider bioturbation that causes significant downward displacement and nanoplastic hotspots along burrow walls.

## Introduction

While plastic pollution has been acknowledged as a major challenge for the marine environment,^1^ recent material flow estimates suggest that comparably more plastic is emitted to soils.^2,3^ Plastics can make their way into soils from diffuse sources, such as mismanaged waste, littering, or as secondary particles from plastic products fragmenting during their use or originating from traffic.^2,4−7^ Agricultural soils are exposed to plastics via application of sewage sludge,^8−11^ compost,^12^ manure,^13^ or other biosolids as soil amendments^14^ and with irrigation water^4^ or when microplastics are released from macroplastics used in agriculture such as mulching films^15^ or packaging material.^16^ Of the emitted plastic, micro- (≤5 mm) and nanoplastics (≤1 μm) are considered problematic due to their potential mobility and susceptibility to ingestion by soil organisms.^5,17−19^ Moreover, there is increasing evidence that these plastic particles can induce changes in soil properties^20−23^ or exert effects on terrestrial microbial communities,^24^ plants,^21,25^ or other soil biota either directly or indirectly.^26−32^

Effects on terrestrial organisms exposed to micro- or nanoplastics are often expressed as a function of average concentrations in the soil, but the smaller scale spatial distribution of contaminants in soils is often more important than average concentrations for their bioavailability and subsequent effects.^20,33^ Our understanding of the terrestrial fate and spatial distribution of microplastics and particularly nanoplastics within the soil profile is still fragmentary,^5,34^ making it challenging to reliably assess the exposure of soil organisms or the long-term fate of these particles. At the same time, the abundance of nonbiodegradable nanoplastics in soils is expected to increase over time as plastic emissions continue and larger particles already present in the soil gradually fragment.^35^ Understanding the spatial distribution and mobility of nanosized plastics in soil will therefore become more important in the future.

Advective transport of nanoplastics with water has been studied comparably well, usually using packed column tests.^36−41^ These tests tend to find low particle mobility when the water content of the soil is low,^42−44^ because particles tend to accumulate at air–water interfaces that are numerous in nonsaturated soils.^45^ However, soils are rarely water saturated, making transport mechanisms other than advective transport potentially more relevant. A transport process that has been largely neglected in terrestrial exposure assessments is bioturbation, the restructuring of the soil by burrowing soil organisms. In particular, the role of deep-burrowing (anecic) earthworms may be important, as they break down and incorporate organic matter in the soil and their burrowing behavior facilitates soil aeration and drainage.^46−49^ In doing so, they contribute to the movement of particles in the soil, not only organic matter^50,51^ but also pollutants sorbed to mineral surfaces or particulate pollutants,^50−53^ including inorganic nanoparticles.^54^

Particle transport by earthworm bioturbation is a combined result of mechanical soil mixing, particles attaching and detaching from the organism surface, and ingestion and excretion dynamics.^52−55^ The contributions of the different bioturbation mechanisms may have implications for the resulting spatiotemporal distribution pattern within the soil profile. Local mixing has been previously used to model transport of engineered nanomaterials and is considered to result in gradual redistribution patterns resembling diffusion.^54,56^ In contrast, ingestion and subsurface excretion may result in longer transport distances at a shorter time span.^57^ First investigations have given qualitative indications that earthworms cause vertical transport of microplastics in the soil profile.^29,53,58^ Earthworms were observed to ingest microplastics^59^ and incorporate them into the soil.^58^ Some authors have suggested nanoplastics would be similarly transported, albeit without providing empirical evidence.^60^ Considering that initial investigations on microplastics showed increasing transport with decreasing size,^53^ it appears reasonable that nanoplastics could be more susceptible to biologically driven transport and, in particular, ingestion/excretion dynamics. However, experimental evidence for nanoplastics uptake by earthworms and biologically mediated transport is still lacking. This is in part due to the challenges associated with the extraction and detection of nanoplastics in materials that contain organic carbon such as soils.^61−63^ Established detection methods for microplastics either omit the nanosized fraction because of size limitations in the case of spectroscopic methods^64,65^ or experience difficulties in detecting small mass concentrations in the case of thermoanalytical methods.^66^

The aim of this study was to assess the impact of earthworms on nanoplastics in soil, with a particular focus on the spatiotemporal dynamics and mechanisms of biologically mediated nanoplastics transport. We hypothesized that nonlocal transport by ingestion and excretion is the main mechanism causing vertical redistribution of nanoplastics in soil profiles. To this end, we performed process studies in microcosms with a deep-burrowing earthworm species, *Lumbricus terrestris*, using a combination of methods that would allow us to more closely understand the mechanisms of biologically mediated transport. We used metal-doped spherical polystyrene nanoplastics to allow for quantitative analysis in soil samples even at dilute concentrations. In a first step, we quantified the time-dependent vertical redistribution of nanoplastics within soil profiles. In a second step, we used X-ray computed tomography (CT) to monitor the earthworm burrow system development and thus mechanistically investigate how bioturbation transports nanoplastics. Finally, the presence of nanoplastics in the drilosphere versus the soil matrix was investigated to explore the potential formation of plastic hotspots following bioturbation. A better quantitative understanding of the transport mechanisms of nanoplastics in soils will provide more accurate estimations of exposure over more extended time periods, which in turn will enable more robust risk assessment and better informed environmental regulation.

## Materials and Methods

### Nanoplastics

We synthesized metal-doped spherical polystyrene nanoplastics, as described in detail by Mitrano et al.,^67^ for investigating transport of nanoplastics by bioturbation. Using the palladium (Pd) label as a proxy to measure the plastic allowed us to more easily measure plastic transport. Pd was incorporated into the center of the particle, so that the surface of the particle was composed entirely of polymer material. Only negligible Pd leaching from these nanoplastic particles was found in experimental systems in previous studies,^10,41,68^ ensuring that Pd is a conservative tracer for nanoplastic particles. Moreover, we consider the particles to remain intact throughout the study period, because polystyrene has negligible biodegradation rates in soils.^69,70^ The content of the metal tracer corresponded to 0.24% w/w of the plastic particles, determined based on dry weight of the nanoplastic suspension (after drying 48 h at 60 °C, *n* = 3) and microwave-assisted *aqua regia* extraction of Pd as described below. A Z-average hydrodynamic diameter of 256 ± 4 nm and a polydispersivity index of 0.096 ± 0.02 (*n* = 12) were found using dynamic light scattering (Malvern Zetasizer Nano ZS), confirming previous measurements on similar batches. Details on particle characterization are provided in the Supporting Information (Supplementary Table S1).

### Soil

We used topsoil from the plough layer of a former agricultural site in Sprowston, UK (WGS 84:387724, 5835408). The soil selection was based on earthworm habitat requirements and the high relevance of plastic pollution for agricultural soils. The soil was classified as a sandy loam (60% sand, 28% silt, 12% clay), with a pH of 7.2–7.6 and 5.0% w/w organic matter. According to the measured soil properties (Table S2), we considered the soil typical for an agricultural plough layer affected by common management practices. The background concentration of Pd in this soil, measured after *aqua regia* digestion, was 32 ± 4 μg kg^–1^ (*n* = 6).

### Earthworms

Adult individuals of the deep-burrowing (anecic) earthworm species *Lumbricus terrestris* were purchased for bioturbation experiments (Wormsdirect, UK). Before being introduced to the microcosms, earthworms were depurated for 48 h. Individuals were rinsed with water, placed in Petri dishes with damp filter paper and kept in the dark at 13 °C to allow them to void their gut. Earthworm casts, i.e., excreted material, were regularly removed to avoid re-eating.^71^ The wet weight of depurated individuals was documented before and after the experiment.

### Bioturbation Microcosms

Two bioturbation experiments were established, addressing the two distinct aims of this study. The first experiment (Exp 1) served to determine the average vertical redistribution of nanoplastics in the soil profile. The second experiment (Exp 2) aimed at shedding light on the associated transport mechanisms by measuring nanoplastics concentrations in the drilosphere versus soil matrix, while also monitoring burrow development. The drilosphere is the soil layer around the burrows, where earthworm activity directly changes the soil structure and composition, and can extend to up to 8 mm from the burrow wall for *L. terrestris*.^72^ Microcosms with earthworms were established in an identical manner in both experiments but sampled differently according to the aims of the study.

The microcosms consisted of polyvinyl chloride (PVC) cylinders (10 cm diameter) packed with moistened soil to a total depth of 30 cm with an average bulk density of 1.24 ± 0.03 g cm^–^^3^ (Figure S1). At the bottom of each cylinder, a thin layer of sand (1 cm) and an aluminum mesh (1 mm mesh size) allowed free water drainage and aeration. A glass-fiber mesh (2 mm) prevented earthworms from escaping through the top. For treatments with plastics, the uppermost 2 cm of soil were spiked with nanoplastics before addition to the soil column. The spiking was done by thoroughly mixing a fraction of the soil with the nanoplastics suspension and then sequentially adding and mixing in the remaining soil. The total nanoplastic concentration for each microcosm was 0.56 g kg^–1^ or 0.06% (equivalent to 10.8 g kg^–1^ or 1.08% in the spiked layer). While these concentrations are relatively high within the spiked layer, they allowed for the detection of nanoplastics in low concentrations when transported into previously uncontaminated soil. Moreover, our concentrations were still lower than in other bioturbation and effect studies^14,28−30,58^ because we aimed to use concentrations which were likely to be present in the environment. Soil moisture was kept at 40%–50% of the water holding capacity corresponding to habitat preferences of earthworms. The water content was maintained via recurring applications of ultrapure water (18.3 mΩ) via spraying, corresponding to an average precipitation of 8.3 ± 1.3 mm per week distributed over two to three application instances (Table S3). Convective transport of nanoplastics either in micro- or macropores is unlikely in these conditions because of the relatively low precipitation rate applied in this study^73^ and unsaturated conditions that enhance particle deposition.^45^ Three depurated earthworms were introduced into each column, corresponding to a density of 382 individuals m^–^^2^. Although this stocking density is relatively high, it is not uncommon for some land uses, such as temperate pastures.^71^ The columns were kept in a growth chamber (CLF Plant Climatics) at 13 °C with 60% relative humidity and continuous daylight (24 h) to minimize the risk of earthworms escaping. A litter layer of oven-dried, crushed leaves (*Tilia cordata*) was added on top as feed at the beginning of the study (4 g). An additional 2 g were added after 2 weeks. For the second experimental set (Exp 2), the leaf litter composition was changed due to seasonal availability (*Fagus sylvatica*).

### Measuring the Average Vertical Redistribution of Nanoplastics in Microcosms (Exp 1)

In the first set of experiments, treatments comprised microcosms with *L. terrestris* and plastic-spiked soil (*n* = 12) and control microcosms with nanoplastics but no earthworms (*n* = 3). At weekly intervals, i.e., after 7, 14, 21, 28 days, three replicate microcosms were destructively sampled by pressing the soil column out of the PVC cylinder and sectioning at depths 0–2, 2–6, 6–15, and 15–29 cm, hereafter referred to as layers 1–4 (Figure S1). The bottom 1 cm of soil was discarded to avoid dilution effects from the sand. We selected a greater vertical resolution at the top of the column since we anticipated a larger change in nanoplastic concentrations closer to the spiked top layer. Control microcosms with added nanoplastics but without earthworms were sampled after 28 days to assess nanoplastics transport induced only by water applications.

### Association of Nanoplastics with Earthworm Burrows (Exp 2)

**Figure 1:**
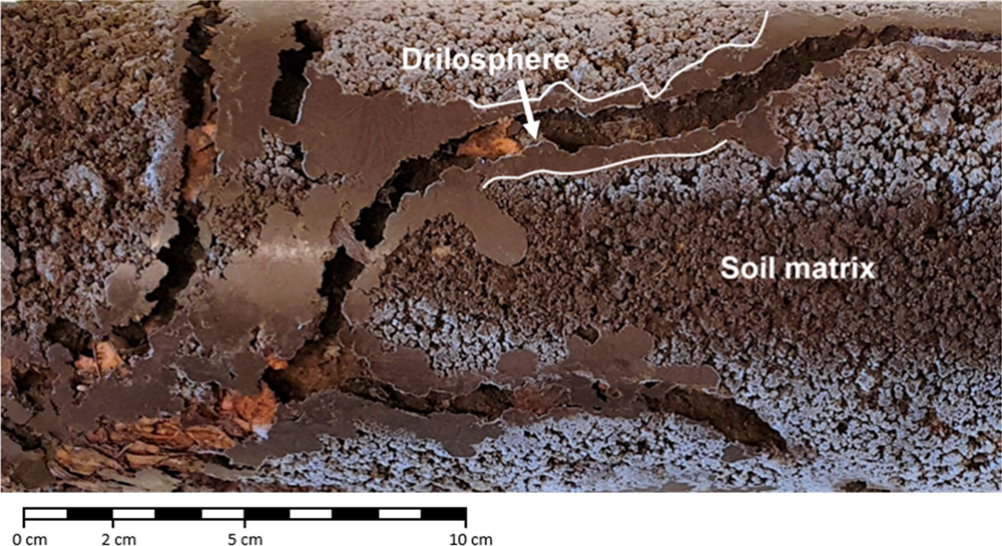
Example of burrows in a soil column after 28 days showing the drilosphere and soil matrix (Exp 2). The different texture of material around burrows is due to casts of earthworms and shows excretion occurs throughout burrows.

During the second set of experiments, X-ray computed tomography (CT) scans were performed weekly to monitor the earthworm burrow system development. Treatments included control microcosms without worms and plastics (*n* = 3), microcosms with *L. terrestris* (*n* = 3), and microcosms with *L. terrestris* and plastics (*n* = 3). After the final X-ray CT scan, i.e., after 28 days, the microcosms were frozen, followed by targeted sampling of the drilosphere and soil matrix to better understand the local spatial distribution of plastics within each sampling layer. Here, soil matrix is defined as being at least 1.5 cm away from any burrow and visually showing no structures indicative of previous earthworm presence. For accessing burrows and unaffected soil matrix, the frozen soil columns were removed from the PVC cylinder. The drilosphere was sampled by carefully scratching off the burrow walls with a metal spoon during thawing at selected sites, where the burrow was intact and accessible, resulting in the analysis of four and three burrows for replicates 1 and 2, respectively. Figure 1 shows an example of the burrow systems, while the exact sampling locations are documented in Figure S2. One replicate was accidentally dropped and destroyed on day 22, so sampling for drilosphere and matrix was only done for two column replicates. The soil matrix samples were taken near the column wall and in the center of the column. The samples were categorized according to the layers they were extracted from.

### X-ray CT Image Acquisition, Processing and Analysis

X-ray CT has been previously successfully applied to determine biopore volume and monitor earthworm burrow development.^54,55,74^ We scanned the microcosms using an industrial X-ray scanner (GE Phoenix v|tome|x 240) in quick scan mode to minimize the radiation exposure of earthworms (see Table S4 for details). The acquired projections were reconstructed into a sequence (image stacks) of cross-sectional images of the column (GE software datos|x, version 2.1). The cross-sectional images (slices) are grids of voxels of 150 μm size, each with a specified gray value that reflects the attenuation of the X-rays by the material present inside a given voxel. As a result, image parts corresponding to specific materials can be extracted, such as air-filled macropores.

Image processing and analyses were carried out using the ImageJ/FIJI software^75,76^ together with the SoilJ plug-in.^77^ First, the imaged columns were moved to the center of the 3-D image canvas, and the coordinates of the PVC wall were detected. The gray values in all horizontal image cross sections were then normalized to standardized values for air-filled pores and the column material.^77^ A joint histogram of the gray values in all 3-D images was compiled on which we determined a joint segmentation threshold as described in Koestel et al.^78^ (Figure S3). The following image segmentation resulted in binary images with voxels assigned to one of the two material classes,^77,79^ i.e., soil pores or soil matrix, the latter also including organic material and earthworms. When earthworms were present inside the burrows in the segmented images, they were manually removed in slice-by-slice editing. For each column, approximately identical regions of interest were analyzed to monitor changes in the earthworm burrow structure across the four measurement instances, with a final average soil column length of 28.6 ± 0.3 cm considered for analysis (0.75 ± 0.34 cm below soil surface, 0.54 ± 0.22 cm cut off at the bottom). Using the PoreSpaceAnalyzer tool in SoilJ,^77^ a 3-D map depicting the pores color coded by diameter was computed and used to filter out pores which were too small to be associated with earthworm burrows. A minimum pore-diameter threshold (≥3.5 mm spherical size) and a volume threshold (≥0.084 cm^3^) were visually determined and applied to all images. We then quantified the burrow volume (cm^3^) per depth layer and in total for each soil column. The respective share of the burrow pore volume per depth layer in relation to the total burrow pore volume of the soil column was then calculated, hereafter referred to as biomacroporosity. This allowed us to compare the spatial distribution of the burrows between the treatments with and without plastics. The visualization of the burrow system was done with the software Drishti (v2.7).^80^ The processing workflow is provided in Figure S4.

### Detection of Nanoplastics in Earthworm Tissue, Soil from Depth Layers, and Drilosphere Samples

Soil and drilosphere samples were oven dried (105 °C, 3 days), homogenized, and subsamples taken in triplicate for analysis by successive halving into half-lots. During the first set of experiments (Exp 1), earthworms collected from columns were rinsed, depurated, and weighed, rinsed again, sacrificed by freezing, and dried in a freeze dryer. Dried earthworm tissue was pretreated with 1.5 mL hydrogen peroxide (H_2_O_2_) overnight. Soils and pretreated earthworm tissue samples were then digested using *aqua regia*(81) in a closed microwave-assisted system (Milestone Ethos Easy, MAXI-44, 80 mL PTFE vessels) following EPA 3051a guidelines.^82^ Pd was measured in digests (diluted 1:10 times for soil samples and 1:5.6 times for earthworm samples) using inductively coupled plasma mass spectrometry (ICP-MS, PerkinElmer Nexion 350D) with a detection limit of 0.09 μg L^–1^ and a quantification limit of 0.29 μg L^–1^ (*n* = 5). The calibration standards were matrix matched to diluted *aqua regia*, and ^115^In was used as an internal standard. Detected Pd concentrations were corrected for procedural blanks and background concentration of the respective matrix (Supporting Information S1). Plastic concentrations were then derived from the measured Pd concentrations using the known Pd-to-plastic ratio. General quality assessment measures, i.e., procedural blanks and spike recoveries, were routinely included. For ensuring detectability of Pd in digests, spike recovery of Pd after digestion was tested in digestion vessels without any matrix (99 ± 8%), and in the presence of soil (83 ± 8%) or earthworm tissue (88 ± 4%). Similarly, the extraction efficiency of plastic-incorporated Pd for the optimized method remained above 91% when spiked into soil (Figure S5). Details on solvents and specifics of the digestion protocols can be found in the Supporting Information S1.

### Statistical Analysis

Two-tailed *t* tests assuming equal variance were run for testing statistical significance of differences between detected plastic concentrations in earthworm tissues and in soil samples across different sampling time points, as well as in drilosphere and soil matrix samples. Additionally, the significance of differences in burrow pore volume in microcosms in the presence versus absence of plastics was tested. Significance tests were executed in Microsoft Excel choosing significance levels of 95%. Nanoplastic mass balances were calculated using the dry weight and the detected nanoplastic concentrations for the respective soil layer in comparison to the initial spike added to each column.

### Modeling Nanoplastic Transport

Vertical nanoplastics transport by bioturbation was modeled using the simple one-dimensional bioturbation model developed by Rodriguez,^83^ which was previously successfully applied for silver sulfide nanoparticles.^54^ The model is described in detail elsewhere,^54,83^ and relevant equations are provided in Supporting Information S2. In brief, the model allows for the prediction of the depth-dependent concentrations for a substance as a function of time based on soil mixing rates. Mixing occurs only between adjacent soil depth segments and is assumed to be proportional to earthworm density. The mixing rate is determined semiempirically using the earthworm density and a fitting parameter that was derived by minimizing the sum of squared differences between experimental and modeled logarithmic concentrations. Thus, vertical transport in the model resembles advective transport but is dependent on earthworm density. Logarithmic concentrations were used to ensure that relatively low concentrations in deeper soil layers had similar weight than higher concentrations in top layers during fitting.

## Results and Discussion

### Earthworms Are Drivers for Significant Nanoplastic Transport in soil

**Figure 2:**
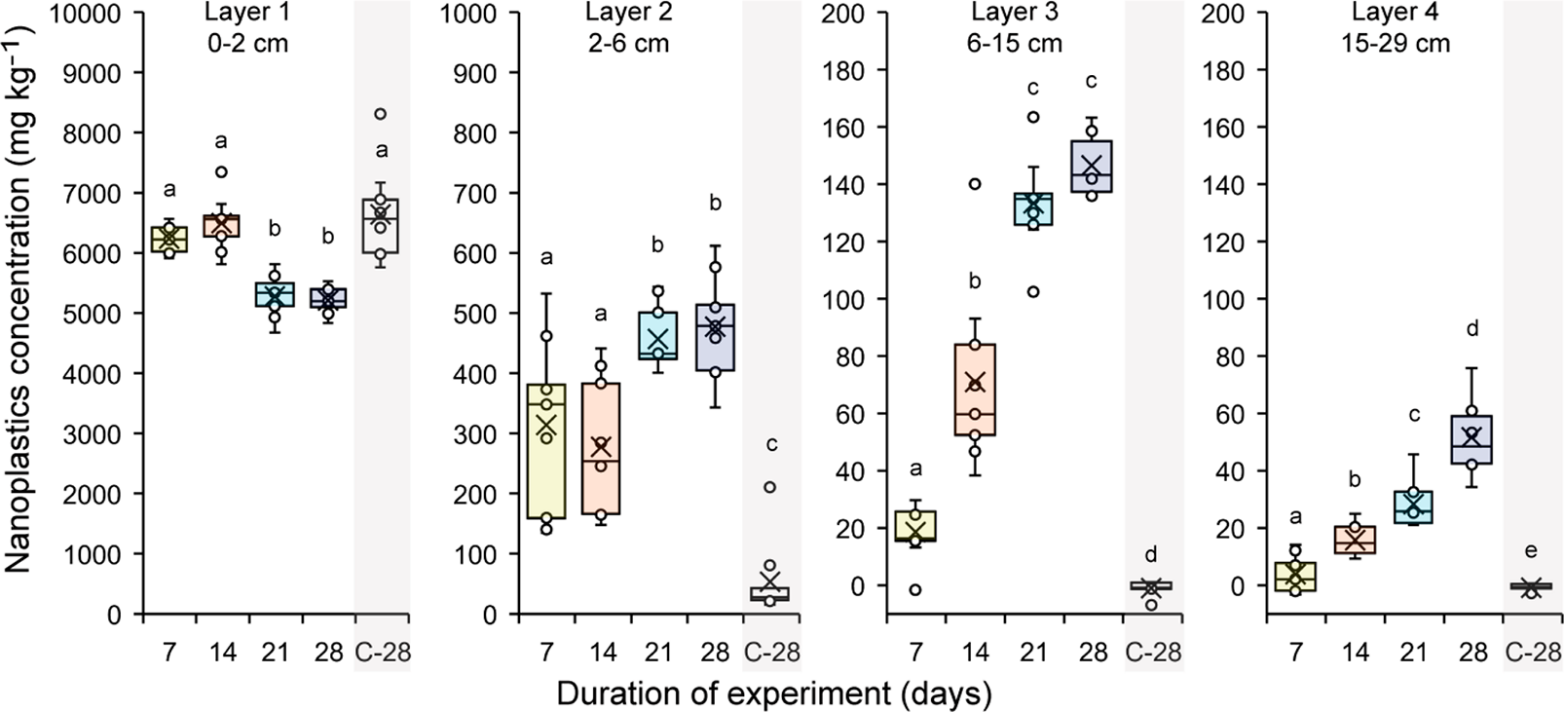
Concentrations of nanoplastics at different soil profile depths across burrowing times by *Lumbricus terrestris* (7, 14, 21, 28 days) and for control columns without *L. terrestris* sampled after 28 days shaded in gray (C-28). Box plots represent the distribution of the first to third quartile. Whiskers display the minimum and maximum (excluding outliers). Points represent individual data points. The lines within the box plots mark the median, and crosses mark the mean. Observations with the same letters do not show significant differences across the respective depth layer (*p* > 0.05).

Deep-burrowing earthworms, *L. terrestris*, were responsible for significant vertical downward transport of nanoplastics in the soil profile (Exp 1, Figure 2). After 1 week, detectable nanoplastic quantities were transported from the uppermost 2 cm of the soil down to the lowest sampling layer. While microcosms in this experiment were limited to 30 cm depth, maximum burrowing depths of *L. terrestris* observed in the field commonly exceed 100 cm.^84^ It is thus reasonable to expect that in field conditions the burrowing of anecic earthworm species such as *L. terrestris* can transport nanoplastics deeper in the soil profile than measured here.

Water applications to the columns were purposefully small to keep the focus on the contribution of bioturbation to nanoplastics transport opposed to advective transport. The soil columns were thus far from water saturated. Preferential flow in macropores is only relevant near water saturation.^85^ Moreover, nanoplastics tend to interact strongly with air–water interfaces limiting their transport in micropores of nonsaturated soils as well.^45^ Accordingly, water-driven nanoplastic transport via earthworm burrows can be considered highly unlikely in our setup. Nanoplastic concentrations in the control columns that were only exposed to water applications without earthworms remained below the Pd background concentration in soil layers deeper than 6 cm even after 28 days of treatment (Figure 2), confirming that, as intended, advective transport was not a major factor in our system. Some nanoplastics were detected in the second layer of these control columns, but these may in part be owed to sampling inaccuracies at the boundary between layer 1, i.e., the initial spike layer, and layer 2. The absence of nanoplastics below 6 cm depth in the control columns is in stark contrast to the nanoplastics measured in deeper soil layers when earthworms were present (Figure 2). Accordingly, it is necessary to explicitly account for bioturbation when investigating nanoplastic fate for unsaturated soils to avoid underestimating their mobility in terrestrial ecosystems.

Our findings on nanoplastics transport complement previous studies that confirmed transport of microplastics (>1 μm) by earthworm burrowing.^53,58^ Previous research was generally limited to single time point measurements, neglecting the temporal dimension of transport processes. In our case, nanoplastics were detectable at a depth of 15–29 cm after the first sampling time point (7 days), and extending time led to higher total concentrations of nanoplastics being transferred into the lower soil profile (Figure 2, Table S5). The absolute share of nanoplastics in the lower two layers increased from 1.3% after 7 days to as much as 11.0% after 28 days (Table S6). Rillig et al. found that 50% of the microplastics (710–850 μm) applied to litter was transferred to depths of 7.0–10.5 cm after 21 days.^53^ The faster mixing observed in their study may be a combined result of spatial confinement, as only 10.5 cm columns were used by these authors, and the fact that the microplastics were applied to litter. These particles were thus more susceptible for ingestion/excretion because litter serves as feeding source for *L. terrestris*.

Interestingly, we observed that the net influx of nanoplastics into layer 3 was not significantly different between 21 and 28 days (*p* > 0.05), while nanoplastic concentrations significantly increased for layer 4 over the course of the experiment. This could indicate that vertical transport of nanoplastics through bioturbation may not necessarily decrease monotonously with depth. These results also illustrate that bioturbation experiments, in general, should not be conducted as one time point measurements and be extended even beyond 4 weeks for capturing potential temporal variations and longer-term trends.

### Earthworms Ingested Nanoplastics, But No Negative Effects Were Observed

Earthworms create their burrows through ingesting soil or moving it mechanically.^49^ In our case, the analysis of depurated earthworms confirmed that nanoplastics initially present in the uppermost 2 cm of the soil were ingested, with some residual plastic found in the gut or tissue of individuals (Table S7). The ingestion and excretion of plastic particles, including nanoplastics, by earthworms have similarly been documented elsewhere.^86−88^ It is unclear whether these nanoplastics were accumulated in tissues or whether they were still present in the gut due to incomplete depuration, but considering that depuration times of *L. terrestris* can extend beyond the 48 h used in this study, the latter is possible.^89^ Earthworms from microcosms that were sampled after 7 days contained higher concentrations of nanoplastics as compared to those sampled in the subsequent weeks (Table S7). During this first week, earthworms needed to establish their burrows, which means they may have spent more time in the top layer of the column where the nanoplastics concentration was highest. In addition, earthworms were starved before the onset of the experiment and were thus more likely to frequent the upper layer to access the leaf litter on the soil surface. Hence, feeding ecology may have contributed to a higher exposure to nanoplastics during the first week of the experiment. Transitions of larger microplastics such as polyethylene beads (710–1400 μm)^53^ or polyester fibers (361 ± 387 μm)^90^ through the gut and excretion by earthworms have been reported. Thus, the decrease of nanoplastics found in earthworm tissue after prolonged experimental time are likely the result of the majority of ingested nanoplastics being excreted again, in conjunction with earthworms ingesting less of the surface layer of soil because burrows were already established. Overall, neither earthworm mortality nor a significant decrease in earthworm weight were noted in this study despite the uptake of nanoplastics and the higher exposure at the onset of the experiment (Table S7). Additionally, no other visible negative effects such as avoidance of the top layer due to nanoplastics contamination were observed as discussed in the following paragraph.

### Earthworm Burrowing Is Not Significantly Altered in the Presence of Nanoplastics

**Figure 3:**
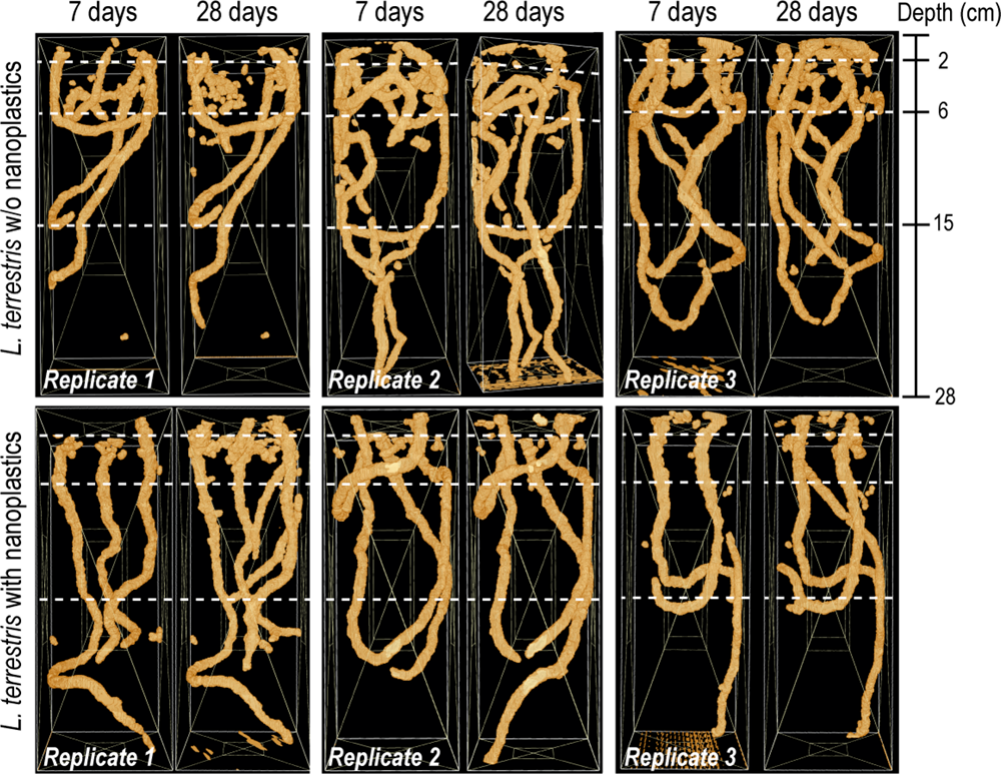
3-D images of the burrow system of *L. terrestris* derived from X-ray CT analysis in experiment 2 (Exp 2) for each replicate column after 7 and 28 days of bioturbation without (top) and in the presence of nanoplastics (bottom). White dotted lines indicate layer boundaries. Note that for replicate 3 with plastics the later image represents 21 days exposure.

**Figure 4:**
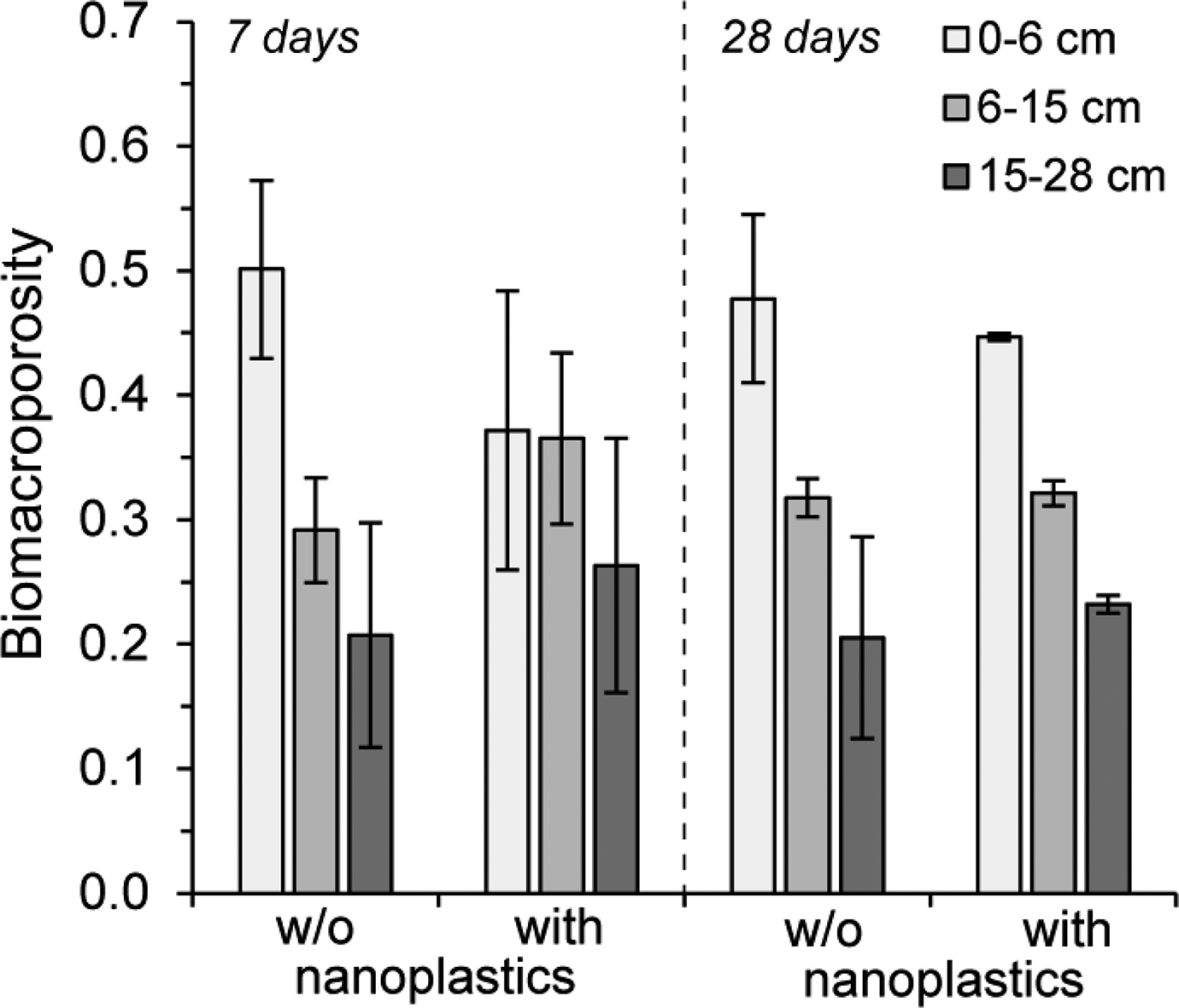
Biomacroporosity in different column segments of experiment 2 after 7 and 28 days of bioturbation without (w/o nanoplastics, *n* = 3) and in the presence of nanoplastics (with nanoplastics, *n* = 3 except after 28 days *n* = 2). Biomacroporosity represents the relative share of the total biopore volume of the respective microcosm soil column for each designated depth layer. The soil column was divided according to sampling layers, with the top two layers merged. Error bars represent standard deviations.

The burrow systems derived from the X-ray CT measurements allowed for the assessment of the activity of *L. terrestris* in the microcosms contaminated by nanoplastics compared to uncontaminated soil (Exp 2, Figure 3). Earthworms established their principal burrow system within the first week, a system that generally remained intact over time, in particular, in the lower part of the soil profile. Over the course of the experiment, existing burrows were expanded, but only a limited number of new burrows were produced (Figure 3). *L. terrestris* are known to inhabit semipermanent burrow systems, and their behavior in the columns typified this.^51^ Irrespective of the treatment, more burrows were created in the upper parts of the soil column. This was confirmed by the spatial distribution of the biomacroporosity within the soil columns as can be seen in Figure 4, with 60%–90% of earthworm burrows present in the upper half of the soil columns (Table S8).

Visual inspection of the burrow system development (Figure 3) suggested that earthworms were overall less active in the presence of nanoplastics, particularly in close vicinity to the areas initially spiked with nanoplastics (0–6 cm column depth, corresponding to layers 1 and 2) at the onset of the experiment. We therefore compared the biomacropores for the different depth segments of the soil profile between the treatments to assess whether there was quantitative evidence to support the avoidance behavior this pattern suggested. This analysis showed that the absolute biomacropore volume was indeed lower in the presence of nanoplastics, a difference that could mostly be attributed to a lower biomacropore volume encountered in the 0–6 cm depth fraction (19 ± 9 cm^3^ with plastics versus 35 ± 8 cm^3^ without plastics) (Table S8). However, the difference between the treatments was not significant (*p* > 0.05), and after prolonged bioturbation (i.e., within 14 days), apparent differences in total burrow pore volume and depth distribution fully disappeared (Figure 4). Moreover, burrow expansion in the presence of nanoplastics during that time was mostly occurring in the uppermost two layers (Table S8).

The absence of active avoidance behavior of nanoplastics is in line with observations from another study that used microplastic fibers in an avoidance-specific assay.^90^ Note that quantifying ecotoxicological effects was not the primary goal of this study, which may have confounded accurate observation of an avoidance behavior. Avoidance-specific assays are typically carried out with contaminated and uncontaminated soils in equal volumes side by side, allowing the organism to move freely between the soils.^90^ At the same time, exposure scenarios in the field are more likely to resemble the approach taken in our study, where the application of plastics would occur at the surface in a shallow layer. Hence, soil biota in the field which typically come to the surface to access food will not necessarily be able to avoid contact with incorporated micro- or nanoplastics, as mimicked in our study.

### Ingestion–Excretion Is the Primary Transport Mechanism Causing Vertical Displacement of Nanoplastics in soils

**Figure 5:**
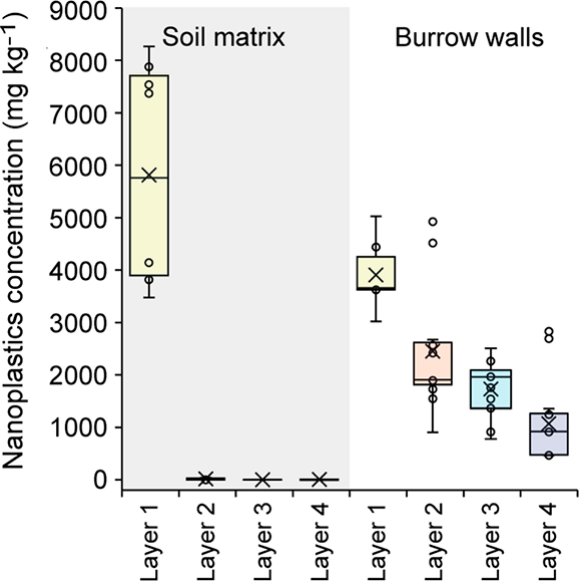
Concentrations of nanoplastics in burrow walls (drilosphere) and unaffected soil matrix at different soil depths after 28 days of soil column exposure to bioturbation by *Lumbricus terrestris*, experiment 2. Results are sorted according to sampling layer. Layer depths correspond to layer 1:0–2 cm, layer 2:2–6 cm, layer 3:6–15 cm, layer 4:15–29 cm. Box plots represent the distribution of the first to third quartile. Whiskers display the minimum and maximum (excluding outliers). Points represent individual data points. The lines within the box plots mark the median, and crosses mark the mean.

Transport via bioturbation is generally a combined effect of local mechanical mixing resulting from the movement of soil during burrowing, ingestion and excretion of material and through the adhesion of particles to the surface of organisms as they move through the soil. However, the individual contributions of these different transport mechanisms may result in different spatial patterns of nanoplastics distribution within the soil profile, in particular, transport distances.^52^ The results of Exp 2 confirm that nanoplastic transport was primarily occurring within the burrows of *L. terrestris*. After 4 weeks, drilosphere samples were analyzed for their nanoplastics concentration in comparison to the soil matrix (Figure 5). Nanoplastics were highly enriched within the drilosphere, whereas concentrations within the soil matrix were orders of magnitude lower and often lower than the Pd background concentration. To our knowledge, this is the first evidence that nanosized plastics can be incorporated into the drilosphere by earthworms, as has been previously observed for microplastics.^58,91^

We infer that nonlocal transport by ingestion and excretion was the dominant mechanism causing the deeper vertical redistribution of nanoplastics in the burrow system for three reasons. First, we confirmed that nanoplastics were ingested and thus also excreted by *L. terrestris*. Second, visual inspection confirmed that casts of *L. terrestris* were incorporated into the drilosphere throughout the burrow system, which were easily distinguishable by their fine texture and density (Figure 1). This reworking of burrow walls with cast material is well documented for *L. terrestris*,^47,92,93^ and similar hotspot-like patterns have been observed for redistributed organic matter by earthworms.^46^ Gut transit times of *L. terrestris* have been estimated at approximately 11.6 h.^94^ Earthworms could thus easily have reached deeper soil layers and excreted ingested material.

Finally, local transport through mixing is unlikely to explain the observed deep vertical transfer that was observed after the relatively short experimental times of 7–28 days. At first glance, the significant decline (*p* ≤ 0.05) of average nanoplastics concentrations in the drilosphere with increasing burrow depth (Figure 5) suggests local transport through the mechanical movement of soil, i.e., mixing. However, our X-ray CT data shows that mixing after 1 week was mostly restricted to the top of the soil columns where burrows were more actively produced (0–6 cm), and correspondingly, more soil material was moved around (Figure 3). In contrast, vertical burrows reaching down to the lower layers remained intact until the end of the experiment, with only limited expansions or additions (Figure 3, Table S8). Nevertheless, plastic concentrations increased continuously in the bottom two layers of the column (Figure 2) despite this limited mixing. We fitted a bioturbation model based on local mixing, previously found suitable for other nanoparticles,^54^ to the observed time- and depth-dependent nanoplastic concentrations. The fitted local transport model correctly estimated the concentrations in the deepest soil layer but systematically overestimated nanoplastic transport to the second and third soil layer (Figure S6, Table S9). In other words, an unusually high soil turnover rate of ca. 25 cm year^–1^ had to be assumed to predict the concentrations in the bottom layer. In comparison, typical turnover rates are in the order of 0.5 cm year^–1^.^83^ The transport of nanoplastics into the deepest soil layer thus proceeds at a faster rate than to the overlying soil layers (Figure S6). As a result, the local mixing model was unable to account for the observed spatial and temporal pattern of nanoplastic transport (Figure S7). Indeed, the distribution of nanoplastics did not always follow a strict depth-dependent trend within an individual burrow crossing different depth segments (Figure S7). In some cases, plastic concentrations in the burrow walls in the lower layer were similar to or exceeded those in the layers above, resulting in small-scale hotspots that indicate nonlocal transport processes. Consequently, ingestion and excretion dynamics are likely the major cause for the deeper vertical transfer that occurred over a timespan as short as 28 days.

### Environmental Implications and Limitations

A better understanding of the temporal and spatial dynamics of fate processes affecting nanoplastics is a crucial step toward assessing long-term exposure levels within soils and also understanding the terrestrial contribution to aquatic plastic pollution. Our results show that ingestion and excretion by earthworms transport nanoplastics to the deeper soil layers in relatively short timespans from 7 to 28 days. While the transport of nanoplastics by bioturbation in our study was limited to only 30 cm depth due to restraints in our experimental setup, their transport will likely expand beyond this depth considering the deep burrowing behavior of *L. terrestris*. These results emphasize the need to account explicitly for bioturbation when characterizing nanoplastic fate in soil, but they also suggest that the dominant bioturbation mechanism is relevant. While we did not compare transport of nanoplastics with microplastics, we hypothesize the dominant bioturbation mechanism to be size specific. Ingestion and excretion of nanoplastics are more likely than for larger microplastics.^53^ Hence, earthworms likely transport higher numbers of nanoplastics and, at least initially, over larger distances.^57^

Similarly, our observations call for a more critical assessment of exposure assessments, in particular, field sampling approaches for quantifying plastic pollution in soils. The most current studies reporting environmental concentrations of microplastics only analyze the uppermost part of the topsoil, ignoring the potential transport of plastic particles within the soil profile. For more accurate mass estimates and balances on larger scales in the future, considering the vertical distribution in monitoring schemes for micro- and nanoplastics is of importance.

While we only used one soil type under constant climatic conditions, it is important to note that field bioturbation rates differ highly between different localities. Bioturbation rates are highly dependent on the number of bioturbating organisms and species. These in turn depend on climatologic circumstances, physical and chemical soil properties, and land management.^95^

The redistribution of nanoplastics may also carry important implications for local exposure levels. Exposure levels to nanoplastics in the field are likely very heterogeneous as we observed that nanoplastics were highly enriched within the drilosphere. Earthworm burrows and the associated drilosphere can thus become hotspots of nanoplastic pollution. Earthworm species such as *L. terrestris* that move repeatedly through the same burrows and reingest burrow material^46,51^ and other organisms that use earthworm burrows and the drilosphere as habitats may be more exposed even if average concentrations within the soil matrix are comparatively lower. Acute negative impacts were not observed in this study, whereas effects of chronic long-term exposure are still unknown.

An enrichment of plastics within the drilosphere may also result in combined effects of water- and bioturbation-driven transport processes. Earthworm burrows serve as pathways for preferential flow of water where flow rates, and thus shear rates, are higher than in micropores.^96^ During heavy rainfall events, preferential flows may more easily remobilize nanoplastics and transport them to deeper soil layers and potentially even to shallow groundwater layers or nearby freshwater systems. Such preferential flows in earthworm burrows have been observed for microplastics in one study.^97^ Similar tests would therefore be useful to gain a better understanding of the remobilization potential of the nanosized plastic fraction to reliably assess the associated risk of plastic transfers.

At the current time, it is still uncertain how different plastic shapes, sizes, earthworm species, or soil properties may affect bioturbation transport dynamics in terms of transport mechanisms and ultimately transport depth and rate. However, our study emphasizes that a more systematic understanding of bioturbation-driven transport of micro- and nanoplastics could not only advance our knowledge on the long-term fate of micro- and nanoplastics and flows between different environmental compartments but could also inform decisions made for in-field measurements and monitoring.

## Supporting Information Available

Nanoplastic characterization and soil properties, microcosm setup and sampling, X-ray CT measurement and workflow, digestion protocol, bioturbation model description and results, earthworm weight and uptake of nanoplastics, sampling procotol for drilosphere sampling, detected nanoplastic concentrations in soil samples (Exp 1) and drilosphere and soil matrix samples (Exp 2) (PDF)
