# Supplementary material for: Nanoplastic Transport in Soil via Bioturbation by Lumbricus terrestris
Source: Environ Sci Technol. 2021 Dec 8;55(24):16423–33. doi: 10.1021/acs.est.1c05614 (PMC8697554; doi:10.1021/acs.est.1c05614)
Supplement: Supplementary file 1 — es1c05614_si_001.pdf [file es1c05614_si_001.pdf]

Supporting information

Nanoplastic Transport in Soil via Bioturbation by *Lumbricus terrestris*

Wiebke Mareile Heinze[a], *Denise M. Mitrano*[b], *Elma Lahive*[c], *John Koestel*[a,d], *Geert Cornelis*[a]

[a]Swedish University of Agricultural Sciences, Department of Soil and Environment, Box 7014, 75007 Uppsala, Sweden

[b]ETH Zurich, Department of Environmental Systems Science, Universitätsstrasse 16, 8092 Zürich, Switzerland

[c]UK Centre for Ecology and Hydrology, Benson Lane, Crowmarsh Gifford, Wallingford, OX10 8BB, UK

[d]Agroscope – Standort Reckenholz, Soil Quality and Soil Use, Reckenholzstrasse 191, 8046 Zürich, Switzerland

Total number of pages: 18

Total number of figures: 7

Total number of tables: 9

List of supplementary materials

Supplementary material	Page
**Supplementary material S1**. Digestion protocol for Pd-doped nanoplastics.	S4
**Supplementary materials S2**. Description of bioturbation model used for simulating nanoplastics transport in soil.	S5

List of tables

Table	Page
**Table S1**. Properties of palladium-doped polystyrene nanoplastics[1] used for quantifying nanoplastics transport by bioturbation in soil, Experiment 1 (Exp 1) and Experiment 2 (Exp 2).	S7
**Table S2**. Selected details including physical and chemical soil properties for the topsoil used in microcosm studies, Exp 1 and Exp 2.	S8
**Table S3**. Average water content (% of water holding capacity) and dry bulk density of soil columns (g cm^{-3}) for each layer Exp 1. Water applications are provided as average (mm per week) for all replicates.	S8
**Table S4**. Measurement parameters and instrument specifications for X-ray computed tomography (CT) scans, Experiment 2 (Exp 2).	S9
**Table S5**. Average nanoplastic concentrations (top) calculated from Pd concentrations measured in the soil profiles of microcosms (bottom), Exp 1. For each layer, triplicate soil samples (Soil rep 1-3) were analyzed. The average Pd background concentration was subtracted (32 μg kg^{-1}).	S9
**Table S6**. Total and per layer recovery of nanoplastics in microcosm soil columns at each timepoint, calculation based on average concentration and weight of soil (dry weight) per layer, Exp 1.	S10
**Table S7**. Earthworm weight, weight change and incorporated nanoplastics concentrations, Exp 1. Values are averages for each microcosm with three individual earthworms. Significance of differences ($p \leq 0.05$) was tested for the depurated wet weight before and after the experiment.	S11
**Table S8**. X-ray CT derived biomacropore volume (cm^3, biopores with ≥3.5 mm spherical diameter) and biomacroporosity (%, share of the total biopore volume inside the respective soil layer) in microcosm soil columns with and without plastics after 7 and after 28 days, Exp 2.	S12

Table S9. Applied parameters used for modelling nanoplastic transport by bioturbation (Exp 1) using the bioturbation model developed by Rodriguez (2006). S12

List of figures

Figure Page

Figure S1. Schematic overview of setup and sampling scheme of microcosm columns for investigating bioturbation induced transport of nanoplastics by *Lumbricus terrestris* (left) and arrangement of columns in the growth chamber (right), Exp 1 and Exp 2. S13

Figure S2. Sampling locations of drilosphere samples in replicate 1 (left, 1) and replicate 2 (right, 2), Exp 2. In total, 7 intact burrows were sampled (B1-B7), and classified according to the depth layer samples were taken from (L1-L4). In one case two samples for layer 4 were analyzed separately (L4.1 and L4.2). S14

Figure S3. Joint histogram of gray-values for all normalized X-ray image sequences to determine a joint segmentation threshold, Exp 2. S15

Figure S4. Detailed workflow of X-ray CT image processing and analysis, Exp 2, including applied tools and corresponding references in ImageJ/Fiji using the SoilJ plug-in. References for the corresponding tools are denoted with letters, a: Koestel (2018), b: Legland *et al.* (2016). S15

Figure S5. Results of optimization of analytical protocol for digesting nanoplastics and extracting incorporated palladium with *aqua regia*: open-vessel hot plate and closed-vessel microwave-assisted digestion, and direct injection of diluted nanoplastics suspension into ICP-MS. The recovery of nanoplastic-incorporated Pd in presence of soil is included for the final method. S16

Figure S6 Experimental and modelled nanoplastics concentrations in the four depth layers (0-2, 2-6, 6-15, 15-28 cm) of Exp 1 using a simple bioturbation model developed by Rodriguez (2006). S16

Figure S7. Nanoplastics concentrations detected in drilosphere (burrow wall) samples (Exp 2), sorted according to burrow number and sampling layer. Highlighted (blue) are burrows from soil column replicate 1, without shading are burrows from replicate 2. Note that for Burrow 7 two separate samples from the lowest layer were analyzed. S17

List of references of supplementary information S18

Supplementary materials

Supplementary materials S1. Digestion protocol for Pd-doped nanoplastics.

Palladium (Pd) doped nanoplastics nanoplastics (0.24% w/w) were used throughout the experiments, where Pd concentrations were determined in *aqua regia* extracts of soil (Exp 1), including drilosphere (Exp 2), and earthworm tissue using inductively coupled plasma mass spectrometry (ICP-MS) as a proxy for nanoplastics concentrations. Soil samples were oven-dried (105°C, 3 days), homogenized and ground by mechanical sieving (Exp 1) or manually in an agate mortar (Exp 2) due to differences in sample size. The bulk sample of each layer was subsampled by dividing the soil into half-lots by pouring the sample carefully over the edge of a container onto an even surface. This procedure was repeated until subsamples of approximately 2-3 g were achieved. For each sample triplicates of approximately 0.5-0.6 g were then transferred to the polytetrafluoroethylene (PTFE) digestion tubes (80 mL) using glass weighing floats. The exact weight of each sample was noted. For earthworms, the freeze-dried tissue was carefully divided into smaller pieces, added to digestion tubes (wich exact weight noted) and treated with 1.5 mL hydrogen peroxide (30 vol.% H_2O_2) overnight.

Aqua regia extraction of soils or earthworm tissue was done in a closed microwave-assisted system (Milestone Ethos Easy, MAXI-44). After sample addition, acids were sequentially applied to each sample: for soils 1.25 mL of 65 vol.% nitric acid (HNO_3) and 3.75 mL of 37 vol.% hydrochloric acid (HCl). For earthworm tissue, with weights of 0.5-1.0 g of dry material we applied a higher total volume composed of 1.875 ml HCl, and 5.625 ml HNO_3. The microwave digestion program followed EPA 3051 guidelines[2]: The temperature was ramped from 20°C to 175°C in 20 minutes (1800 W for > 9 vessels), kept constant at 175°C for 10 minutes and then cooled down for at least 50 minutes, or until temperatures were below 25°C. *Aqua regia* extracts were then transferred quantitatively to 50 mL volumetric flasks over a funnel and filter (Munktell filter paper) through repeatedly rinsing the tube with ultrapure water (18.3 Ω). Both filter and

funnel were then rinsed with ultrapure water to dilute the sample to a total volume of 50 mL, resulting in 10 times diluted extracts for soils and drilosphere, and 5.56 times for earthworms. The Pd-concentrations in the *aqua regia* extract were analyzed by inductively coupled plasma mass spectrometry (ICP-MS) (Perkin Elmer, Nexion 350D) with a detection limit of 0.09 µg L^{-1} (n=5). Pd-concentrations detected in the extracts were corrected for procedural blanks of the respective extraction set and for the respective background concentrations of Pd in the matrix. In case of soil samples, this was 32 ± 4 µg kg^{-1} (n=6) and for earthworm tissue this was 0.016 ± 0.012 µg kg^{-1} (n=3).

The extraction efficiency of plastic-incorporated Pd was optimized by testing different methods and digestion systems (Figure S5): 1) *aqua regia* digestion in an open-vessel hotplate system (n=3), 2) *aqua regia* digestion in a closed-vessel microwave-assisted system (n=3), 3) direct injection of diluted nanoplastic suspension (n=3). The highest extraction efficiencies were observed for the closed-vessel microwave-assisted *aqua regia* digestion (Figure S5), which was subsequently used for all further analysis throughout the experimental work.

Supplementary materials S2. Description of bioturbation model used for simulating nanoplastics transport in soil.

The bioturbation model is a one-dimensional mathematical model which was developed by Rodriguez[3], and was applied here as has previously been done by Baccaro et al.[4]. The bioturbation rate constant (equation 1) was retrofitted by minimizing the sum of squared errors between the logarithm of experimental time- and depth dependent concentrations and calculated ones based on equation 2. For this equation, the soil profile is divided into discrete depth layers of length d_l (m). Concentrations C (mg kg^{-1}) are changed only by mixing between directly adjacent layers ($l+1$ and $l-1$). The bioturbation rate is thus a semi-empirical parameter, proportional to a soil turnover rate

$v_{l:l+1}$ (m s^{-1}) that is proportional to earthworm density and dependent on a bioturbation fitting parameter β (m^4 s^{-1}) (Equation 3).

$$k_{bioturb,l:l+1} = \frac{v_{l:l+1}}{d_l} \qquad \text{(Equation 1)}$$

$$[C]_{l,t+x} = [C]_{l,t} + k_{bioturb,l:l+1,t}\,\delta t\big([C]_{l+1,t} - [C]_{l,t}\big) + k_{bioturb,l-1:l,t}\,\delta t([C]_{l-1,t} - [C]_{l,t})$$

(Equation 2)

$$v_{l:l+1} = \beta w_l \qquad \text{(Equation 3)}$$

$k_{bioturb,l:l+1}$: bioturbation rate (s^{-1})

$v_{l:l+1}$: soil turnover rate (m s^{-1})

d_l: depth of layer with an associated concentration (m)

[C]: concentration of substance (mg kg^{-1})

δt: time-step considered for model (s^{-1})

β: bioturbation fitting parameter (m^4 s^{-1})

w_l: earthworm density (individuals m^{-3})

The parameters applied for this study are shown in Table S9. Note that for obtaining average concentrations for the soil depth layers of different thickness as investigated in our study, the simulated concentrations for the corresponding model depth layers were summed together.

Supplementary Tables

Table S1. Properties of palladium-doped polystyrene nanoplastics[1] used for quantifying nanoplastics transport by bioturbation in soil, Experiment 1 (Exp 1) and Experiment 2 (Exp 2).

Nanoplastics properties									
**Particle material**									
Shell material		Polystyrene							
Core material		Polyacrylonitrile							
Shape		Spherical							
Surface morphology		Raspberry-shell							
Suspension solution		Traces of SDS from synthesis process							
Palladium content		0.24 %							
**Particle size and charge**									
Z-average diameter[a]	nm	256	±4			*n=12*			
PDI[a]		0.10	±0.02			*n=12*			
Zeta potential in ultrapure water[b]	mV	-60	±1			*n=3*			
Zeta potential in soil leachate[b]	mV	-38	±1			*n=3*			
**Dilution series**									
Particle concentration	mg L^{-1}	170		85		34		17	
Z-average diameter[a]	nm	255	±2	254	±6	258	±2	258	±3
PDI[a]		0.12		0.10		0.09		0.08	
**Time series**									
Time	days	0		6		15		35	
Z-average diameter[a]	nm	255	±2	254	±5	251	±6	251	±4
PDI[a]		0.12		0.07		0.07		0.07	

[a]*Measurement conditions: 3 replicates, 13 readings per replicate, 18 °C, 120 s equilibration time, signal-to-noise >0.9, >600 kcps, attenuator value 6-7*

[b]*Electrophoretic mobility calculated into Zeta potential using the Smoluchowski approximation*

Table S2. Selected details including physical and chemical soil properties for the topsoil used in microcosm studies, Exp 1 and Exp 2.

Soil characterization			
Land use history		Agricultural	
Soil type		Topsoil	
Location	Sprowston, Norfolk, UK	WGS84: 387724, 5835408	
Soil texture		Sandy loam	
Coarse fragments	2-20 mm	3	%
Sand	2.0-0.063 mm	60	%
Silt	0.063-0.002 mm	28	%
Clay	<0.002 mm	12	%
Water holding capacity		420	mL kg^{-1}
Palladium concentration		32 ± 4	mg kg^{-1}
pH		7.2-7.6	
Soil organic matter		5.0	w/w %

[a]The English/Welsh soil classification uses 0.06 mm as a boundary between sand and silt[5]

Table S3. Average water content (% of water holding capacity) and dry bulk density of soil columns (g cm^{-3}) for each layer for Exp 1. Water applications are provided as average (mm $week^{-1}$) for all replicates.

Parameter	Sampling layer	7 days		14 days		21 days		28 days		Control (28 days)	
Water content at sampling timepoint (% of WHC)	Layer 1	40	±1%	41	±3%	49	±2%	45	±0%	42	±1%
	Layer 2	42	±2%	43	±2%	46	±1%	46	±3%	41	±2%
	Layer 3	40	±2%	42	±2%	41	±1%	42	±4%	42	±2%
	Layer 4	36	±1%	36	±1%	35	±1%	35	±2%	36	±1%
Bulk density at sampling timepoint (g cm^{-3})	Layer 1	0.93	±0.14	0.98	±0.08	0.99	±0.08	0.99	±0.08	1.13	±0.07
	Layer 2	1.07	±0.09	1.14	±0.09	1.19	±0.08	1.25	±0.17	1.13	±0.02
	Layer 3	1.20	±0.12	1.21	±0.07	1.26	±0.02	1.28	±0.02	1.20	±0.01
	Layer 4	1.34	±0.04	1.29	±0.07	1.26	±0.07	1.23	±0.01	1.29	±0.06
Average water application per week (mm)		7.7	±0.7	8.8	±0.6	9.8	±1.4	7.0	±0.6	8.3	±1.3

Table S4. Measurement parameters and instrument specifications for X-ray computed tomography (CT) scans, Exp 2.

Measurement parameter		
Volt	150	kV
Current	700	µA
Exposure time per projection	250	ms
Number of projections	2000	
Average	1	
Images skipped	0	
Binning	2 x 2	
Voxel size	150	µm
Multi-scan	Yes	
Fast scan	Yes	

Table S5. Average nanoplastic concentrations (top) calculated from Pd concentrations measured in the soil profiles of microcosms (bottom), Exp 1. For each layer (layer 1-4, here L1-L4), triplicate soil samples (Soil rep 1-3) were analyzed. The average Pd background concentration was subtracted (32 µg kg^{-1}).

			Microcosm replicate 1			Microcosm replicate 2			Microcosm replicate 3					
			Soil rep 1	Soil rep 2	Soil rep 3	Soil rep 1	Soil rep 2	Soil rep 3	Soil rep 1	Soil rep 2	Soil rep 3	**Mean**	**Stdev**	**RSD**
**Nanoplastic concentrations (mg kg^{-1})**	7 days	L1	6554	5913	6023	6222	6566	6313	6419	5993	6108	6235	± 244	4%
		L2	159	140	135	533	462	381	292	373	348	314	± 144	48%
		L3	16.4	15.5	13.2	25.7	24.5	15.5	<bkg	29.7	27.7	18.5	± 9.7	52%
		L4	<bkg[a]	<bkg	<bkg	14.1	12.1	7.7	2.1	7	<bkg	3.9	± 6.5	280%
	14 days	L1	7344	6562	6398	6012	5814	6814	6614	6609	6279	6494	± 448	7%
		L2	441	245	254	148	166	164	383	412	284	278	± 112	40%
		L3	52	47	60	38	84	55	93	140	70	71	± 31.3	44%
		L4	11	10	9	14	15	15	20	25	22	15.7	± 5.6	34%
	21 days	L1	4675	4933	5123	5339	5358	5619	5117	5498	5814	5275	± 353	7%
		L2	424	425	447	432	543	400	537	405	501	457	± 55	12%
		L3	137	146	135	130	124	102	163	126	137	133.3	± 16.6	12%
		L4	33	29	21	33	21	46	22	26	25	28.3	±7.9	28%
	28 days	L1	5529	5302	5394	4836	5419	5099	5113	4988	5201	5209	± 223	4%
		L2	343	404	402	612	510	514	478	576	459	478	± 86	18%
		L3	n.a.[b]	n.a.	n.a.	136	163	136	142	158	144	146.6	± 11.6	8%
		L4	n.a.	n.a.	n.a.	76	61	53	34	42	44	51.6	± 15.0	29%
	Control (28 days)	L1	7170	8313	6671	5983	5758	6005	6888	6418	6569	6641	± 775	12%
		L2	81	211	43	28	23	21	25	33	15	53	± 62	117%
		L3	<bkg	<bkg	<bkg	<bkg	<bkg	0.9	0.9	1.1	<bkg	<bkg	± 2.4	-241%
		L4	<bkg	<bkg	<bkg	0.6	<bkg	<bkg	0.4	<bkg	0.3	<bkg	± 1.3	-138%

Table S5 (continued).

			Microcosm replicate 1			Microcosm replicate 2			Microcosm replicate 3				
			Soil rep 1	Soil rep 2	Soil rep 3	Soil rep 1	Soil rep 2	Soil rep 3	Soil rep 1	Soil rep 2	Soil rep 3	**Mean**	**Stdev**
**Pd concentrations (µg kg^{-1})**	7 days	L1	15757	14215	14479	14958	15786	15176	15432	14408	14685	14989	± 586
		L2	383	337	324	1280	1110	915	702	898	838	754	± 346
		L3	39.3	37.3	31.7	61.8	59.0	37.2	<bkg	71.4	66.6	44.5	± 23
		L4	<bkg	<bkg	<bkg	34.0	29.0	18.5	4.9	16.9	<bkg	9.3	± 26
	14 days	L1	17655	15775	15382	14453	13976	16381	15901	15888	15097	15612	± 1078
		L2	1060	590	610	356	400	395	921	990	683	667	± 268
		L3	125.8	112.3	143.7	92.2	201.8	132.0	223.6	336.8	167.7	170.7	± 75
		L4	27.0	23.6	22.2	32.8	37.1	35.3	48.8	60.0	53.1	37.8	± 13
	21 days	L1	11238	11859	12316	12837	12881	13509	12301	13218	13978	12682	± 848
		L2	1018	1022	1075	1040	1307	963	1290	973	1204	1099	± 133
		L3	328.3	350.9	324.2	312.2	298.4	246.3	392.9	302.7	328.6	320.5	± 40
		L4	78.3	68.8	50.7	79.3	50.8	109.9	52.5	62.2	61.0	68.2	± 19
	28 days	L1	13292	12748	12967	11626	13028	12258	12292	11991	12504	12523	± 537
		L2	824	972	966	1471	1225	1235	1150	1386	1103	1148	± 208
		L3	n.a.	n.a.	n.a.	326.3	392.2	326.7	341.2	381.0	347.2	352.4	± 28
		L4	n.a.	n.a.	n.a.	182.3	146.3	127.8	82.5	101.3	104.8	124.2	± 36
	Control (28 days)	L1	17239	19985	16038	14383	13842	14437	16560	15430	15793	15967.4	± 1864
		L2	194	507	103	66	54	51	60	79	35	127.8	± 150
		L3	<bkg	<bkg	<bkg	<bkg	<bkg	2.2	2.2	2.6	<bkg	<bkg	± 6
		L4	<bkg	<bkg	<bkg	1.5	<bkg	<bkg	1.0	<bkg	0.7	<bkg	± 3

[a] Lower than the background Pd-concentration of the soil;
[b]Not available

Table S6. Total and per layer recovery of nanoplastics in microcosm soil columns at each time point, calculation based on average concentration and dry weight of soil per layer, Exp 1.

Layer ID	Depth	Recovery of spiked plastic (%) 7 days	14 days	21 days	28 days	Control – 28 days
Layer 1	0-2 cm	56 %	61 %	50 %	50 %	72 %
Layer 2	2-6 cm	6.5 %	6.0 %	10.5 %	11.3 %	1.2 %
Layer 3	6-15 cm	1.0 %	3.8 %	7.3 %	8.1 %[a]	0.0 %
Layer 4	15-29 cm	0.3 %	1.4 %	2.4 %	2.9 %[a]	0.0 %
Total	0-29 cm	64 %	73 %	70 %	72 %	74 %
**Layer ID**	**Depth**	**Recovery of spiked plastic (mg) 7 days**	**14 days**	**21 days**	**28 days**	**Control – 28 days**
Layer 1	0-2 cm	913.5	999.7	815.4	807.4	1177.7
Layer 2	2-6 cm	106	97.6	171.1	184.7	18.9
Layer 3	6-15 cm	15.6	61.5	118.6	132.3[a]	0.1
Layer 4	15-29 cm	5.5	22.2	39.2	46.5[a]	0
Total	0-29 cm	1040.6	1181	1144.2	1170.9	1196.7

[a]Based on triplicate samples from two replicates only

Table S7. Earthworm weight, weight change and incorporated nanoplastics concentrations, Exp 1. Values are averages for each microcosm with three individual earthworms. The significance of differences ($p \leq 0.05$) was tested for the depurated wet weight before and after the experiment.

Time (days)	Replicate	Initial worm weight wet (g)[a]	Final worm weight wet (g) [a]	Change in weight (g)	Change in weight (%)	t-test, p-values	Worm weight dry (g dw)[b]	Average plastic (µg/g dw)[c]	RSD (%)
7	R1	6.7 ±1.1	6.2 ±0.5	-0.6	-8.3	0.47	1.2 ±0.3	1135 ±776	68
	R2	6.1 ±0.8	4.8 ±0.3	-1.4	-22.1	0.52	0.9 ±0.1	1048 ±628	60
	R3	5.3 ±0.3	5.1 ±1.1	-0.3	-4.7	0.32	0.7 ±0.0	n.a.	
Average[d]		6.0 ±0.9	5.4 ±0.8	-0.7	-11.7		1.0 ±0.2	1090 ±633	58
14	R1	5.5 ±0.5	6.5 ±1.2	1.0	17.5	0.25	0.8 ±0.2	120 ±90	73
	R2	6.3 ±0.7	6.4 ±0.9	0.0	0.1	1.00	0.9 ±0.1	260 ±176	67
	R3	6.7 ±0.1	7.8 ±0.3	1.1	16.2	0.01	1.1 ±0.1	380 ±272	71
Average		6.2 ±0.7	6.9 ±1	0.7	11.3		0.9 ±0.2	253 ±202	80
21	R1	6.2 ±0.5	6.5 ±0.8	0.3	4.0	0.65	0.9 ±0.1	211 ±143	67
	R2	6.3 ±1.0	7.1 ±0.7	0.8	13.4	0.31	0.9 ±0.0	153 ±101	66
	R3	5.3 ±0.9	5.7 ±0.9	0.5	8.6	0.56	0.7 ±0.1	234 ±83	35
Average		5.9 ±0.9	6.4 ±0.9	0.5	8.7		0.8 ±0.1	199 ±103	52
28	R1	5.8 ±0.8	5.5 ±0.3	-0.3	-4.9	0.61	0.8 ±0.1	311 ±142	45
	R2	6.0 ±0.5	6.1 ±0.5	0.1	2.1	0.76	0.9 ±0.1	574 ±363	63
	R3	5.8 ±0.9	5.8 ±1.1	0.0	0.1	1.00	0.8 ±0.2	276 ±254	91
Average		5.9 ±0.7	5.8 ±0.7	-0.1	-0.9		0.9 ±0.1	387 ±272	70

[a]Depurated during 48 h
[b]After freeze-drying
[c]Corrected for background concentration in blank earthworm tissue
[d]Results based on worms extracted from two replicates (n=6)

Table S8. X-ray CT derived biomacropore volume (cm³, biopores with ≥3.5 mm spherical diameter) and biomacroporosity (%, share of the total biopore volume inside the respective soil layer) in microcosm soil columns with and without plastics after 7 and after 28 days, Exp 2.

Treat-ment	Time (days)	Repli-cate (nbr)	Total	Layers 1 and 2 (0-6 cm)		Layer 3 (6-15 cm)		Layer 4 (15-30 cm)	
			Biomacroporevolume (cm³) and biomacroporosity (%)						
			(cm³)	(cm³)	(%)	(cm³)	(%)	(cm³)	(%)
without (w/o) nano-plastics	7	1	53	30	57 %	17	33 %	5	10 %
		2	86	44	51 %	21	24 %	21	25 %
		3	72	31	43 %	22	30 %	20	27 %
		Mean	70	35	50 %	20	29 %	15	21 %
		Stdev	17	8	7 %	2	4 %	9	9 %
without (w/o) nano-plastics	28	1	62	34	54 %	21	33 %	8	12 %
		2	82	33	41%	25	31 %	23	29 %
		3	83	40	48 %	26	31 %	17	20 %
		Mean	76	36	48 %	24	32 %	16	20 %
		Stdev	11.6	3.8	7 %	2.7	2 %	7.9	8 %
with (w) nano-plastics	7	1	55	18	32 %	17	31 %	20	37 %
		2	57	29	50 %	19	34 %	9	16 %
		3*	39	12	29 %	17	44 %	10	26 %
		Mean	50	19	37 %	18	37 %	13	26 %
		Stdev	10	9	11 %	1	7 %	6	10 %
with (w) nano-plastics	28	1	85	38	45 %	27	31 %	20	24 %
		2	65	29	44 %	21	33 %	15	23 %
		3 [a]	53	21	39 %	22	41 %	10	20 %
		Mean	75	34	45 %	24	32 %	17	23 %
		Stdev	13.7	6.4	0 %	3.6	1 %	3.7	1 %

[a] Replicate 3 with nanoplastics was accidentally destroyed after 21 days, and not considered for the calculation of mean or standard deviation

Table S9. Applied parameters used for modelling nanoplastic transport by bioturbation (Exp 1) using the bioturbation model developed by Rodriguez (2006)[3].

Model parameter	Applied values		
Bioturbation rate	$k_{bioturb,l:l+1}$	(s^{-1})	1.59×10^{-5}
Soil turnover rate	$v_{l:l+1}$	($m\ s^{-1}$)	7.95×10^{-8}
Depth of layer with an associated concentration	d_l	(m)	0.005
Initial concentration of substance in top layer	$[C]$	($mg\ kg^{-1}$)	10.84
Time-step considered for model	δt	(s)	1210
Bioturbation fitting parameter	β	($m^4\ s^{-1}$)	5.83×10^{-11}
Earthworm density	w_l	(individuals m^{-3})	1364

Supplementary Figures.

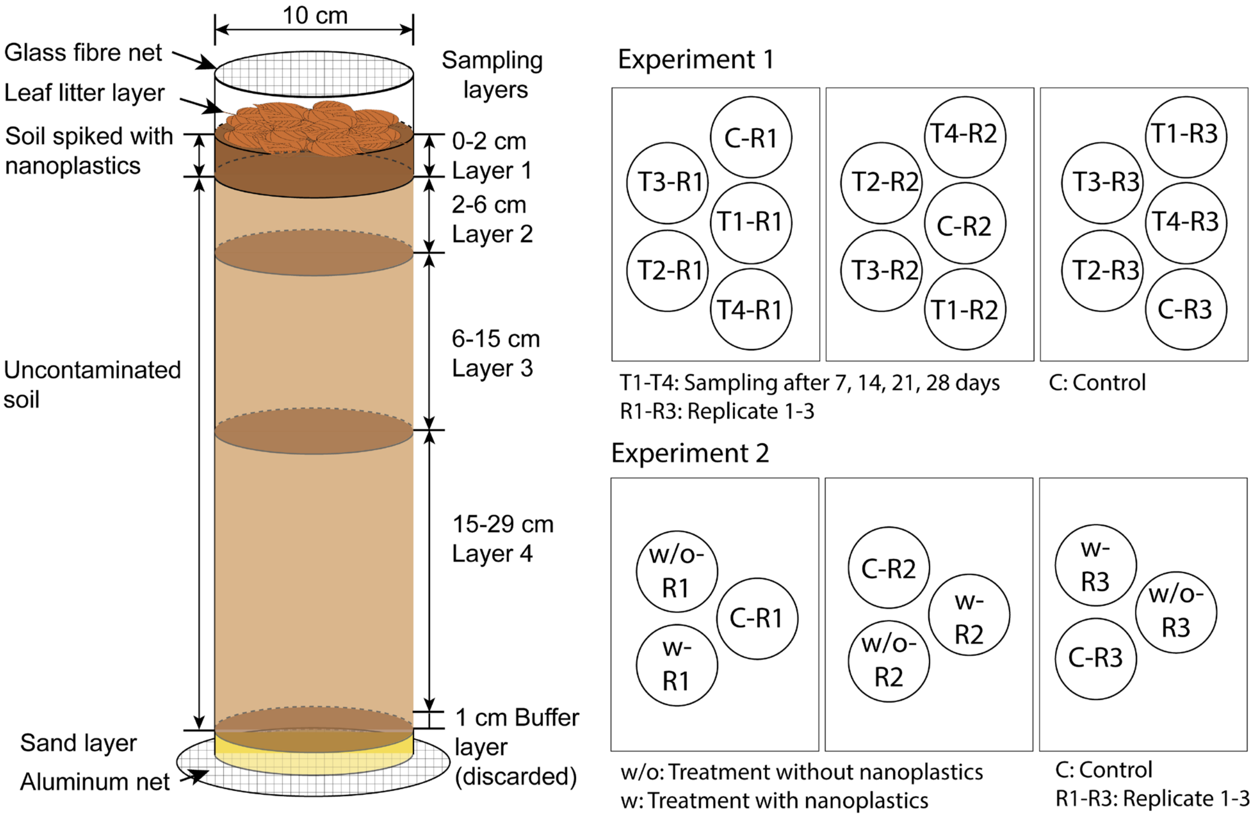

Figure S1. Schematic overview of setup and sampling scheme of microcosm columns for investigating bioturbation-induced transport of nanoplastics by *Lumbricus terrestris* (left) and arrangement of columns in the growth chamber (right), Exp 1 and Exp 2.

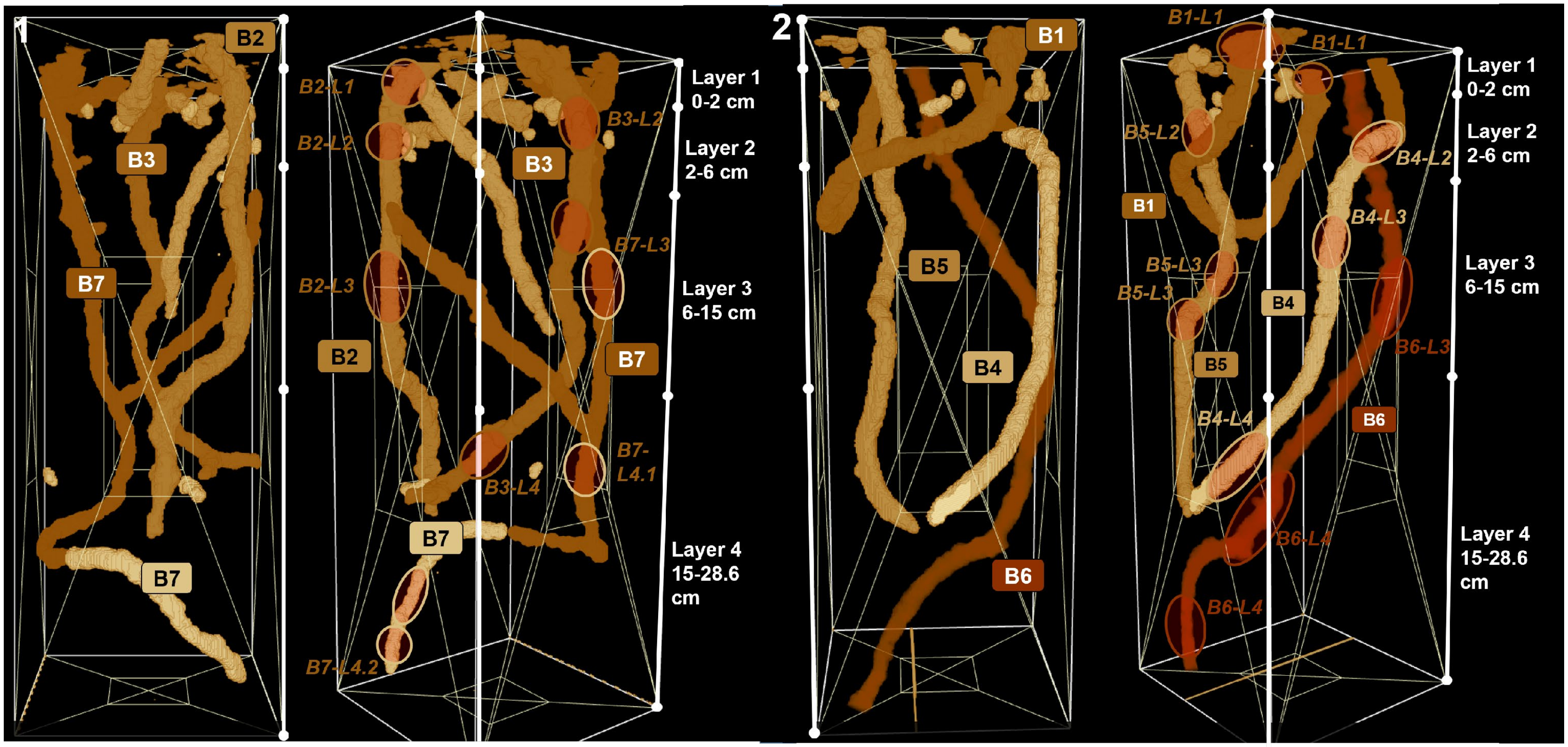

Figure S2. Sampling locations of drilosphere samples in replicate 1 (left, 1) and replicate 2 (right, 2), Exp 2. In total, 7 intact burrows were sampled (B1-B7), and classified according to the depth layer samples were taken from (L1-L4). In one case two samples for layer 4 were analyzed separately (L4.1 and L4.2).

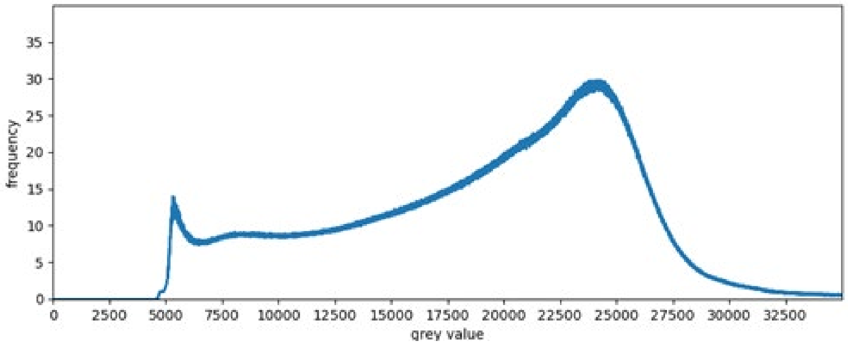

Figure S3. Joint histogram of gray-values for all normalized X-ray image sequences to determine a joint segmentation threshold, Exp 2.

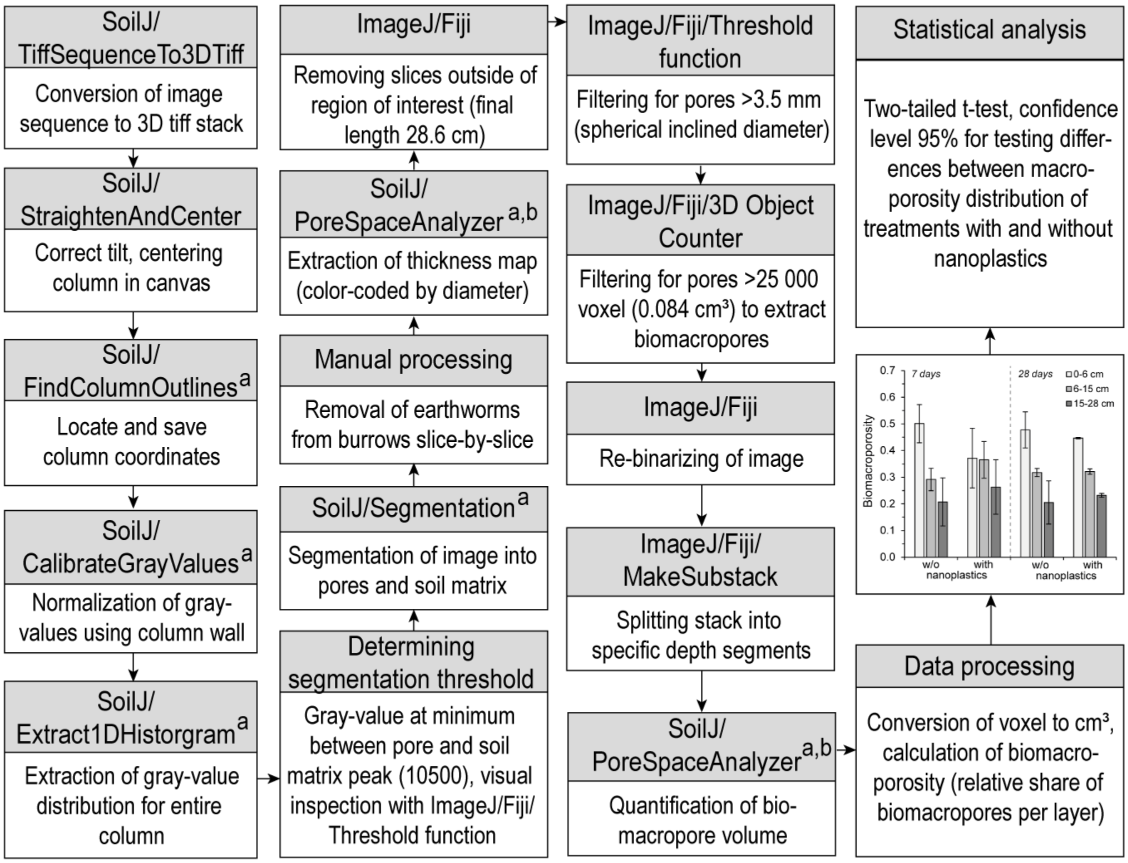

applied tools and corresponding references in ImageJ/Fiji using the SoilJ plug-in. References for the corresponding tools are denoted with letters, a: Koestel (2018)[5], b: Legland, *et al.* (2016)[6].

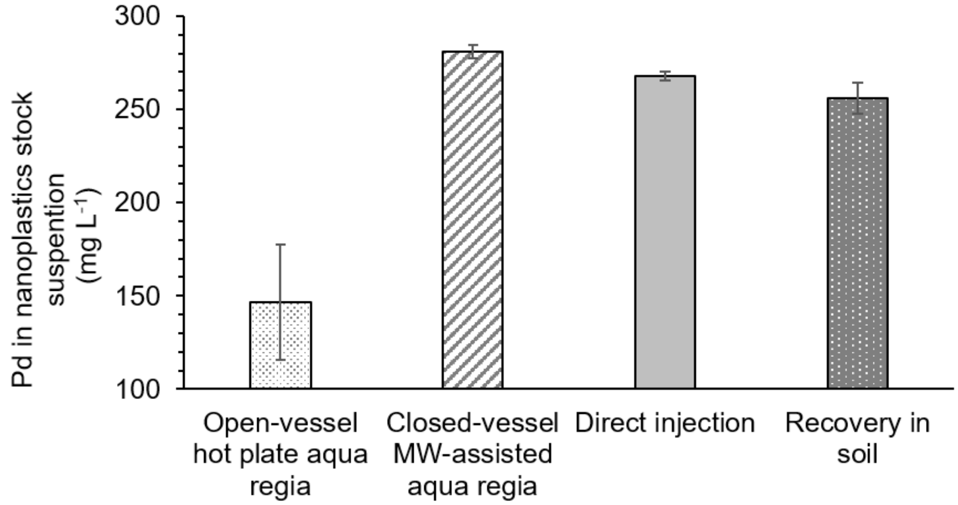

incorporated palladium with *aqua regia*: open-vessel hot plate and closed-vessel microwave-assisted digestion, and direct injection of diluted nanoplastics suspension into ICP-MS. The recovery of nanoplastic-incorporated Pd in presence of soil is included for the final method.

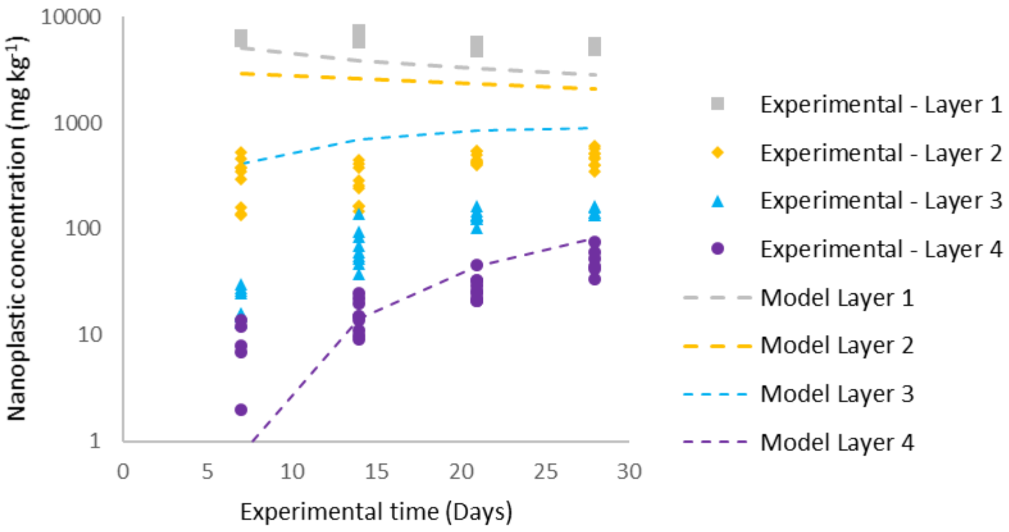

Figure S6. Experimental and modelled nanoplastics concentrations in the four depth layers (0-2, 2-6, 6-15, 15-28 cm) of Exp 1 using a simple bioturbation model developed by Rodriguez (2006)[3].

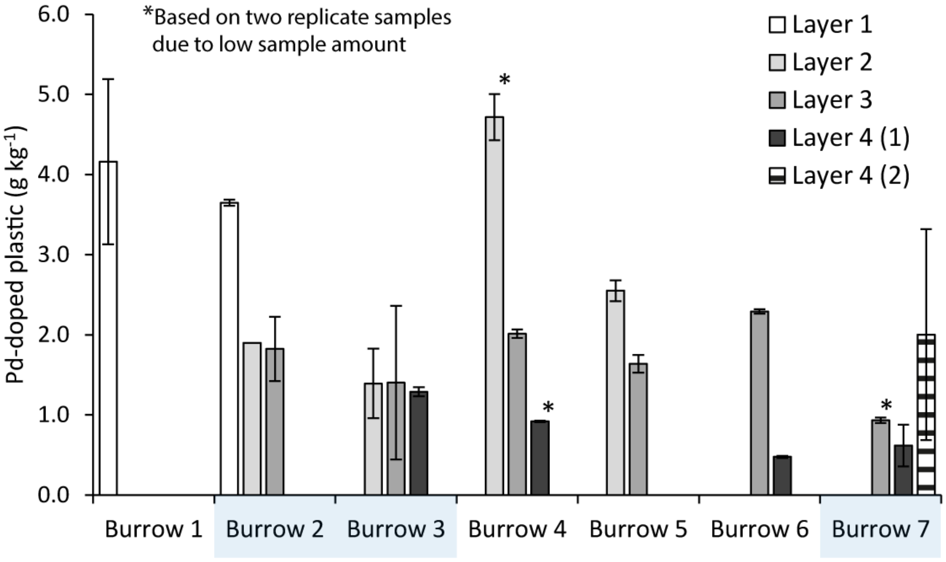

2), sorted according to burrow number and sampling layer. Highlighted (blue) are burrows from soil column replicate 1, without shading are burrows from replicate 2. Note that for Burrow 7 two separate samples from the lowest layer were analyzed.

References for supplementary information

(1) Mitrano, D. M.; Beltzung, A.; Frehland, S.; Schmiedgruber, M.; Cingolani, A.; Schmidt, F. Synthesis of Metal-Doped Nanoplastics and Their Utility to Investigate Fate and Behaviour in Complex Environmental Systems. *Nat. Nanotechnol.* **2019**, 9. https://doi.org/10.1038/s41565-018-0360-3.

(2) U.S. EPA. Method 3051A (SW-846): Microwave Assisted Acid Digestion of Sediments, Sludges, and Oils. **2007**, No. Revision 1.

(3) Rodriguez, M. D. The Bioturbation Transport of Chemicals in Surface Soils, Louisiana State University, 2006.

(4) Baccaro, M.; Harrison, S.; Berg, H. van den; Sloot, L.; Hermans, D.; Cornelis, G.; Gestel, C. A. M. van; Brink, N. W. van den. Bioturbation of Ag2S-NPs in Soil Columns by Earthworms. *Environ. Pollut.* **2019**, *252*, 155–162. https://doi.org/10.1016/j.envpol.2019.05.106.

(5) Martín, M. Á.; Pachepsky, Y. A.; García-Gutiérrez, C.; Reyes, M. On Soil Textural Classifications and Soil-Texture-Based Estimations. *Solid Earth* **2018**, *9* (1), 159–165. https://doi.org/10.5194/se-9-159-2018.

(6) Koestel, J. SoilJ: An ImageJ Plugin for the Semiautomatic Processing of Three-Dimensional X-Ray Images of Soils. *Vadose Zone J.* **2018**, *17* (1), 170062. https://doi.org/10.2136/vzj2017.03.0062.

(7) Legland, D.; Arganda-Carreras, I.; Andrey, P. MorphoLibJ: Integrated Library and Plugins for Mathematical Morphology with ImageJ. *Bioinformatics* **2016**, *32* (22), 3532–3534. https://doi.org/10.1093/bioinformatics/btw413.
